# First Guatemalan record of natural hybridisation between Neotropical species of the Lady’s Slipper orchid (Orchidaceae, Cypripedioideae)

**DOI:** 10.7717/peerj.4162

**Published:** 2017-12-22

**Authors:** Dariusz L. Szlachetko, Marta Kolanowska, Fred Muller, Jay Vannini, Joanna Rojek, Marcin Górniak

**Affiliations:** 1 Department of Plant Taxonomy and Nature Conservation, The University of Gdańsk, Gdańsk, Poland; 2 Department of Biodiversity Research, Global Change Research Institute AS CR, Brno, Czech Republic; 3 Unaffiliated, Ciudad de Guatemala, Guatemala; 4 Department of Plant Cytology and Embryology, The University of Gdańsk, Gdańsk, Poland; 5 Department of Molecular Evolution, The University of Gdańsk, Gdańsk, Poland

**Keywords:** *Cypripedium*, Cypripediaceae, Hybridization, ENM analysis, Nuclear markers, Taxonomy, *Irapeana*

## Abstract

The first natural hybrid in the section *Irapeana* of the orchid genus *Cypripedium* is described and illustrated based on Guatemalan material. A molecular evaluation of the discovery is provided. Specimens with intermediate flowers between *C. irapeanum* and *C. dickinsonianum* within ITS and *Xdh* sequences have the signal sequence of both these species. The analysis of plastid sequences indicated that the maternal line is *C. irapeanum*. Information about the ecology, embryology and conservation status of the novelty is given, together with a distribution map of its parental species, *C. irapeanum* and *C. dickinsonianum*. A discussion of the hybridization between *Cypripedium* species is presented. The potential hybrid zones between the representatives of *Cypripedium* section *Irapeana* which were estimated based on the results of ecological niche modeling analysis are located in the Maya Highlands (*C. dickinsonianum* and *C. irapeanum*) and the eastern part of Southern Sierra Madre (*C. molle* and *C. irapeanum*). Moreover, all three *Cypripedium* species could inhabit Cordillera Neovolcánica according to the obtained models; however, it should be noticed that this region is well-distanced from the edges of the known geographical range of *C. molle*.

## Introduction

*Cypripedium* L. species are found throughout the subtropical to temperate latitudes of the northern hemisphere, excluding northern Africa (Cribb, 1997; Perner, 2008; Eccarius, 2009). The genus has the widest distribution range of all genera included in the subfamily Cypripedioideae. The section *Irapeana* initially included three Mesoamerican species and one Californian endemic, *C. californicum* A. Gray (Cribb, 1997). Later, González Tamayo & Ramírez (1992) described a new species within the group, namely *C. luzmarianum* R. González & R. Delgad., based on a collection from the Jalisco-Michoacán border, but according to Cribb & Soto-Arenas (1993) it represents only a part of morphological variation of *C. irapeanum* Llave & Lex*.* More recently, González Tamayo & Hernández Hernández (2010) segregated three more species from *C. irapeanum*. All these new entities are endemic to the Mexican States of Colima, Jalisco, Michoacan and Nayarit.

Numerous discriminative characters were provided by the authors during publication of *C. susanae* R. González & L. Hernández, *C. gomezianum* R. González & L Hernández and *C. conzattianum* R. González & L Hernández, e.g., plant habit (caespitose vs solitary), length/width/depth ratio of the lip, size and shape of petals, density of pubescence of petals and staminode, size of staminode and stigma, form of trichomes, and habitat. It is noteworthy that some differences between their species are very subtle, which can be seen in the key to determination. The question remains open as to whether they deserve the status of separate species or if the observed differences fall within the infraspecific variation of broadly distributed *C. irapeanum*. From the species proposed by González Tamayo & Hernández Hernández (2010), at least *C. luzmarianum* and *C. susannae* appear to constitute a discontinuum with *C. irapeanum*.

In its floral characters, *C. californicum* resembles representatives of the section *Obtusipetala.* Liu et al. (in Frosch & Cribb, 2012) proposed a section *Californica* to accommodate this species. More recently, molecular studies presented by Li et al. (2011) and Guo et al. (2012) showed that *C. californicum* is not closely related to Mesoamerican representatives of section *Irapeana.* These three species, *C. irapeanum, C. dickinsonianum* and *C. molle,* possess a prominent, acute, staminodial shield which is ciliate or pubescent at the base. Their lip is adorned with numerous semitransparent windows.

*Cypripedium irapeanum* was described in 1825 by la Llave and Lexarza based on a specimen collected “from the mountains of Irapeo near the city of Morelia” in the Mexican State of Michoacán (Cribb, 1997). *Cypripedium dickinsonianum* was published by Hágsater (1984) based on collections made by the expatriate American artist and plantsman, Stirling Dickinson, from near Comitán in the State of Chiapas, México. Both species were originally believed to have different geographical ranges, with *C. irapeanum* having a relatively wide distribution extending from northwestern Mexico to northern Honduras (*vide* Skinner), including much of the central Guatemalan highlands at elevations up to 3,000 m a.s.l. In contrast, *C. dickinsonianum* was originally described as an endemic from the uplands of eastern Chiapas, México. Soto-Arenas & Solano-Gómez (2007a) and Soto-Arenas & Solano-Gómez (2007b) revised the distribution of both species in Mexico in 2007, and the botanist Mario Véliz discovered these two yellow-flowered *Cypripedium* growing in sympatry in north central Guatemala (Dix & Dix, 2000) and vouchered his collections (BIGU). A recent commentary published on the internet by an anonymous Honduran orchid enthusiast has provided photographic evidence that *C. dickinsonianum* also appears to occur in numbers at one locality in central Honduras (E Mo, pers. comm., 2000, BIGU vouchers deposited by M Véliz). Across the regions of northern Mesoamerica where these two *Cypripedium* species occur, their presumptive geographical and altitudinal ranges overlap significantly. There are, however, only a handful of localities (to date, all located in central and western Guatemala) where they are documented to occur in mixed colonies or in close proximity.

Until recently, no intermediate forms between *C. irapeanum* and *C. dickinsonianum* have been recorded. Based on Hágsater (1984) statement that *C. dickinsonianum* is self-pollinated, Cribb (1997) expressed the opinion that it is unlikely that both species hybridise. In fact the two species differ in flower size (Table 1)—in *C. irapeanum* they are twice as large as in *C. dickinsonianum*. However, there are several cases where species with a greater than two-fold difference in flower size hybridise (Bateman & Farrington, 1987; Bateman & Hollingsworth, 2004). Autogamy is a common phenomenon observed in species colonizing a new habitat, where there are no potential pollinators or where gene flow between individuals is limited. We find it hard, however, to accept Hágsater (1984) finding of autogamy in *C. dickinsonianum*, because this opinion was formulated on the basis of the observation of the number of capsules in plants grown in greenhouse conditions. Hágsater (1984) did not, however, observe pollinia on the stigma. Subsequent observations of cultivated *C. dickinsonianum* in 2002 and 2003 indicated that both autogamous (Cribb & Syrlak Sandison, 1998) and open-pollinated individuals occur within at least one known population in Guatemala. The embryology of *C. dickinsonianum* as well as *C. irapeanum* has not been described, although some data about flower and seed production are given (see Hernández-Apolinar et al., 2012 and references cited therein).

**Table 1:** Comparison of *Cypripedium dickinsonianum*, *C. irapeanum*, *C.*×*fred-mulleri* and *C. molle*.

	*C. irapeanum*	*C.*×*fred-mulleri*	*C. dickinsonianum*	*C. molle*
Plant	100 cm, densely coarsely hairy	<75 cm, densely hairy	<30 cm, densely hairy	22–60 cm, densely hairy
Leaves	<20, 5–18 × 2–6 cm, ovate to ovate-lanceolate, acute to acuminate	<15, 3–8 × 2.8–3.8 cm, ovate to ovate-lanceolate, acute	9–16, 2.5–7 × 1–2 cm, narrowly oblong-lanceolate, acute to acuminate	<18, 3–13 × 2–5 cm, elliptic to lanceolate, acute
Inflorescence	<40 cm, <12-flowered	15–33 cm, 5–8-flowered	3–9 cm, 2–6-flowered	<15 cm, <5-flowered
Floral bract	3–10 cm	4–6.6 cm	2.5–5 cm	to 10 cm
Dorsal sepal	3.4–6 × 2–3.5 cm, elliptic, acuminate	3–3.8 × 1.7–2 cm, elliptic, acute	1.4–2 × 1–1.2 cm, elliptic, acute	2.9–3.4 × 1.5–1.8 cm, elliptic, apiculate
Synsepal	3–6 × 2–3 cm, oblong-elliptic, bifid	2.2–3.2 × 1.6–2 cm, elliptic, obtuse, bifid to completely separated	1.4–2.1 × 0.7–0.9 cm, oblong-elliptic, subobtuse, sometimes furculate	2.5–2.9 × 1.6–1.9 cm, elliptic, sometimes bifid at apex
Petal	4.8–7 × 2.3–3 cm, oblong-elliptic, acute	3.4–4.3 × 1.6–2.1 cm, oblong-elliptic, obtuse	1.9–2.5 × 0.9–1 cm, elliptic-lanceolate, obtuse	3.2–3.8 × 1.7–2.4 cm, elliptic, acute
Lip	4–7 × 3.5–4.5 cm, obovoid-globose, windows all over the surface	3.5–4 × 2.8–3 cm, obovoid-globose, small windows all over the surface	1.9–2.5 × 1–1.2 cm, obovoid, large windows all over the surface	2.4–3.4 × 1.7–2.4 cm, obovoid, windows all over the surface
Staminode	1–1.5 × 0.9–1.2 cm cordiform to trullate, with long, tapering apicule, acute to apiculate	1.2 × 0.7–0.8 cm, trullate, acute	0.5–0.7 × 0.7 cm, transversely elliptic to cordiform, shortly apiculate	0.7–0.9 × 0.7–0.9 cm, suborbicular, shortly apiculate
Distribution	Mexico, Guatemala, Honduras	Guatemala (Alta Vera Paz)	Mexico (Chiapas), Guatemala, Honduras	Mexico (Oaxaca, Puebla)

In 2008 the senior author of this paper received a set of color photographs of a multiflowered *Cypripedium* species taken by Fred Muller, an orchid enthusiast from Guatemala. Mr Muller suspected that he had discovered a naturally-occurring hybrid within a sympatric population of *C. irapeanum* and *C. dickinsonianum*, and provided detailed images of this plant*.* Since its discovery, both *C. irapeanum* and *C. dickinsonianum* continue to maintain stable populations at this particular locality. However, a lot of individuals of the putative hybrid have been found flowering every year since their discovery. Last year, we had the opportunity to study the gross morphology of the mixed population and molecular markers to ascertain the true nature of this presumed new *Cypripedium* taxon from central Guatemala. A method of detecting the hybrid origin of the species is to compare the sequences of the nuclear genomes, which are biparentally inherited with sequences derived from the plastid and mitochondrial genomes. For most angiosperms, including orchids, these genomes are inherited *via* the maternal line. Conflict between the sequences derived from the nuclear and organellar genomes may indicate a hybrid origin for the species. In the case of young hybrids, the introgression is low and the analysis of nuclear sequences should show a signal of both parental species. Regardless of the degree of introgression, plastid sequences are derived from one parent—the receiver of the pollen (seed parent). The aim of our study was to ascertain whether morphologically intermediate individuals were hybrids between *C. irapeanum* and *C. dickinsonianum* and to estimate the location of potential hybrid zones.

## Methods

### Ecological niche modeling analysis

**Figure 1:**
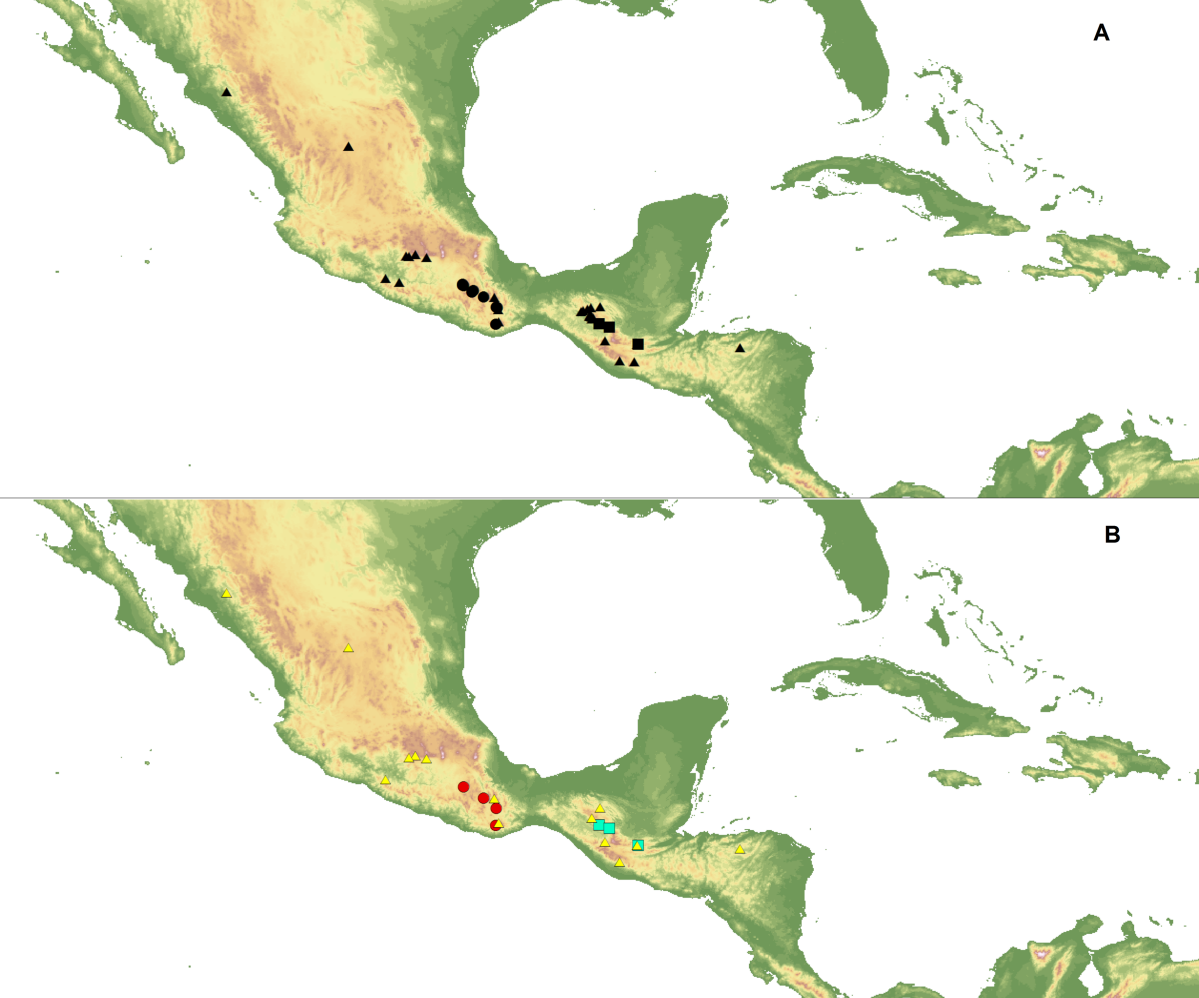
Localities of *C. dickinsonianum* (square), *C. irapeanum* (triangle) and *C. molle* (circle) used in ENM analysis. All gathered data (A). Dataset with reduced sampling bias (B). Map generated in QGIS 2.2.0 (QGIS Development Team, 2016).

Ecological niche modeling (ENM) analysis was used to define areas of potential hybridization between *C. dickinsonianum*, *C. irapeanum* and *C. molle* Lindl*.* The modeling was based on the maximum entropy method implemented in Maxent version 3.3.2 (Phillips, Dudík & Schapire, 2004; Phillips, Anderson & Schapire, 2006; Elith et al., 2011) based on species presence-only observations. The area of the analysis extended from −119.940 to −81.155 longitude and from 29.786 to 8.871 latitude. The list of localities was prepared based on information provided in herbarium specimens deposited in the following herbaria: AMES, AMO, BIGU, CAS, DS, herb. Hinton, K, LL, MEXU, MO, MSC, and WTU. In total, 27 georeferenced records for *C. irapeanum*, nine for *C. molle* and only three for *C. dickinsonianum* were gathered. Two datasets of localities were created (Fig. 1). The first included all the assembled data. To reduce sample bias, we applied spatial filtering in the second data set (Boria et al., 2014) and randomly removed localities that were within 25 km of one another, while retaining the most localities possible. With this approach the second dataset included 14 records for *C. irapeanum*, four for *C. molle* and three for *C. dickinsonianum.* Both datasets are provided as Supplemental Information.

Two groups of bioclimatic variables in 2.5 arc minutes (±21.62 km^2^ at the equator) developed by Hijmans et al. (2005) were used, together with the altitudinal data (Table 2). The first group included all 19 variables. From the second dataset, we removed seven “bioclims”, due to their significant and mutual correlation (above 0.9) as evaluated by the Pearson correlation coefficient calculation computed using ENMTools v1.3. The following variables were excluded from the dataset: bio6, bio7, bio9, bio10, bio11, bio16 and bio17.

**Table 2:** Variables used in the modelling.

Code	Variable
bio1	Annual mean temperature
bio2	Mean diurnal range = mean of monthly (max temp–min temp)
bio3	Isothermality (bio2/bio7) (* 100)
bio4	Temperature seasonality (standard deviation *100)
bio5	Max temperature of warmest month
bio6	Min temperature of coldest month
bio7	Temperature annual range (bio5–bio6)
bio8	Mean temperature of wettest quarter
bio9	Mean temperature of driest quarter
bio10	Mean temperature of warmest quarter
bio11	Mean temperature of coldest quarter
bio12	Annual precipitation
bio13	Precipitation of wettest month
bio14	Precipitation of driest month
bio15	Precipitation seasonality (coefficient of variation)
bio16	Precipitation of wettest quarter
bio17	Precipitation of driest quarter
bio18	Precipitation of warmest quarter
bio19	Precipitation of coldest quarter
Alt	Altitude

Initially, four models were created for each studied species. The first one was created based on all known localities of the studied species and all climatic variables. In the second, a reduced dataset of variables was used. The third included records with a reduced sampling bias and all variables. The last model was created based on the same locality dataset and a reduced variable dataset. In all these analyses the maximum number of iterations was set to 10,000 and the convergence threshold to 0.00001. The “random seed” option, which provided a random test partition and a background subset for each run, was applied. The run was performed as a bootstrap with 1,000 replicates, and the output was set to logistic. All operations on GIS data were carried out using ArcGis 9.3 (ESRI; https://www.esri.com/en-us/home) and QGIS applications.

Furthermore, to reduce overfitting (Radosavljevic & Anderson, 2014) of the models resulting from the small sample size, two additional analyses were made. In both these experiments the reduced locality and variables datasets were used with the same setting as described above. In the first study the regularization multiplier was set at 2 and in the second study it was set at 4.

The evaluation of the models was performed using the most common metric - area under the curve (AUC), which was automatically calculated by the MaxEnt application. The niche overlap between the three studied species was calculated using ENMTools v1.3.

### Macromorphological features

Observations *in situ* have been conducted since 2008. The herbarium material was prepared according to standard classical taxonomy procedure and studied using a stereomicroscope. The comparative research was conducted at the following herbaria: AMO, BIGU, MA, P, W and UGDA. The following vegetative characters of individual plants were analyzed: stem (height, surface), leaves (number, size, shape), inflorescence (length, number of flowers), floral bracts/pedicellate ovary ratio, perianth segments (size and surface of tepals and lip), as well as gynostemium (size and shape of the staminodial shield).

### DNA extraction, amplification, sequencing and sequences analysis

Total genomic DNA was extracted from 20 mg of silica-dried petals (Chase & Hills, 1991) from *C*. *irapeanum* (two specimens), the putative hybrid (two specimens), *C*. *molle* (one specimen), and C. *dickinsonianum* (two specimens) using a DNA Mini Plant (A&A Biotechnology, Gdynia, Poland), following the manufacturer’s protocol. The voucher for all specimens is Fred Muller *s.n*., Guatemala, BIGU. The nuclear ribosomal region spanning the internal transcribed spacers (ITS1 and ITS2) and the 5.8S rRNA gene (ITS), nuclear low copy gene *Xdh* and plastid gene *mat*K were used for detection of the hybrid origin of specimens from Guatemala. ITS was amplified using the primers 17SE and 26SE (Sun et al., 1994). X*dh* was amplified using the primers Xp551F and Xp1590R (Górniak et al., 2014). The gene *mat*K was amplified with the following two primers: - 19F (Molvray, Kores & Chase, 2000) and 1326R (Cuénoud et al., 2002). Polymerase chain reactions (PCR) were carried out in a total volume of 25 µl, containing 5 µl 5× buffer, 1 µl 50 mM MgCl_2_ (only plastid markers), 1 µl 5mM dNTPs, 0.5 µl of 10 µM of each primer, 1 µl dimethyl sulfoxide (DMSO) (only ITS and *Xdh*) and 1.0 unit of Blue Perpetual DNA polymerase (Eurx, Gdansk, Poland). Amplification conditions for ITS and *mat*K were 94 °C for 4 min; 30× (94 °C, 45 s; 52 °C, 45 s; 72 °C, 1 min/2 min, respectively); and 72 °C, 7 min. A touchdown protocol was used for PCR amplification of the *Xdh*: the initial denaturation step (94 °C for 2 min) was followed by six cycles of 94 °C for 45 s, 55 °C (reducing 1 °C per cycle) for 45 sand 72 °C for 90 s. The next 28 cycles used 94°C for 45 s, an annealing step at 49 °C for 45 s, and 72 °C for 90 s. The final extension step used 72 °C for 5 min. PCR products were purified using a High Pure PCR Product Purification Kit (Roche Diagnostic GmbH, Mannheim, Germany). Cycle sequencing was performed using a Big Dye Terminator v 3.1 Cycle Sequencing Kit (Applied Biosystems Inc., ABI, Warrington, Cheshire, UK) with the same primers as were used for PCR amplification: 2.0 µl of 5× sequencing buffer, 1.0 µl of Big Dye Terminator with 1.5 µl of 1 µM primer, 1–4 µl of amplified product (30–90 ng/µl), and 0.5 µl DMSO and H_2_O in a total of 10 µl reaction volume. Cycle sequencing conditions were as follows: 25 cycles each with 15 s denaturation (94 °C), 5 s annealing (52 °C) and 4 min elongation (60 °C). The sequences were generated on an ABI 3720 automated capillary DNA sequencer from Genomed LLC (Warszawa, Poland). Both strands were sequenced to assure accuracy in base calling. Finch TV (Geospiza) was used to edit the sequences, and the two complementary strands were assembled using AutoAssembler (ABI). Representatives of the sections of the genus *Cypripedium* (gene *matK*) were downloaded from GenBank: JQ182208 *Cypripedium molle*, JQ182205 *Cypripedium debile*, JQ182207 *Cypripedium irapeanum*, AF263649 *Cypripedium calceolus*, AY557208 *Cypripedium calceolus*, JQ182204 *Cypripedium acaule*, JQ182203 *Cypripedium palangshanense*, JQ182202 *Cypripedium margaritaceum*, JQ182206 *Cypripedium subtropicum*, JQ182201 *Cypripedium californicum*, JQ182200 *Cypripedium passerinum*, JQ182199 *Cypripedium candidum*, JQ182198 *Cypripedium farreri*, JQ182197 *Cypripedium tibeticum*, JN181460 *Cypripedium fasciculatum*, JN181459 *Cypripedium bardolphianum*, JN181458 *Cypripedium japonicum*, JN181457 *Cypripedium flavum* and EF079360 *Selenipedium aequinoctiale.* All sequences were aligned by eye using SeaView v. 4 (Gouy, Guindon & Gascuel, 2010). For detection of seed parent plastid data (*matK*) the matrix was analyzed using the PAUP* heuristic search method (Phylogenetic Analysis Using Parsimony *and Other Methods) version 4.0b10 (Swofford, 2002). The optimality criterion was the likelihood of tree-bisection-reconnection (TBR) branch swapping and the MULTREES option was in effect. The internal support of clades was evaluated by the bootstrap (Felsenstein, 1985) method with 500 replicates. The General Time Reversible model of substitution with gamma distribution (GTR+G) was selected as the best fitting model by Akaike information criterion in ModelTest v. 3.7 (Posada & Crandall, 1998). To show hybridization visual pairwise comparisons were made (ITS, *XDH*).

### Embryological study

Four capsules from dry material were tested to assess the developmental stages of the ovules/seeds. The procedure of staining in tetrazolium chloride was used (TTC; Van Waes & Debergh, 1986, modified; M Rykaczewski, pers. comm., 2017). After pretreatments (10% glucose, 24 h; then 1% of sodium hypochlorite solution, pH 7.5, 30 min;) the pieces of placenta with ovules/seeds were incubated in 1% TTC in phosphate buffer (pH 7.5) at 40 °C for 24 h. The analyses of pieces were performed firstly under a stereomicroscope (Nikon SMZ 1500) and then examined under a Nikon Eclipse E 800 microscope equipped with differential interference contrast (DIC) optics.

The developmental stages were assessed for approx. 500 ovules/ seeds of each capsule (100 randomly selected ovules, 5 repeats).

### Journal nomenclatural statement

The electronic version of this article in Portable Document Format (PDF) will represent a published work according to the International Code of Nomenclature for algae, fungi, and plants (ICN), and hence the new names contained in the electronic version are effectively published under that Code from the electronic edition alone. In addition, new names contained in this work which have been issued with identifiers by IPNI (International Plant Names Index) will eventually be made available to the Global Names Index. The IPNI LSIDs can be resolved and the associated information viewed through any standard web browser by appending the Life Science Identifier (LSID) contained in this publication to the prefix “http://ipni.org/”. The online version of this work is archived and available from the following digital repositories: PeerJ, PubMed Central, and CLOCKSS.

## Results

### Ecological niche modeling analysis

**Figure 2:**
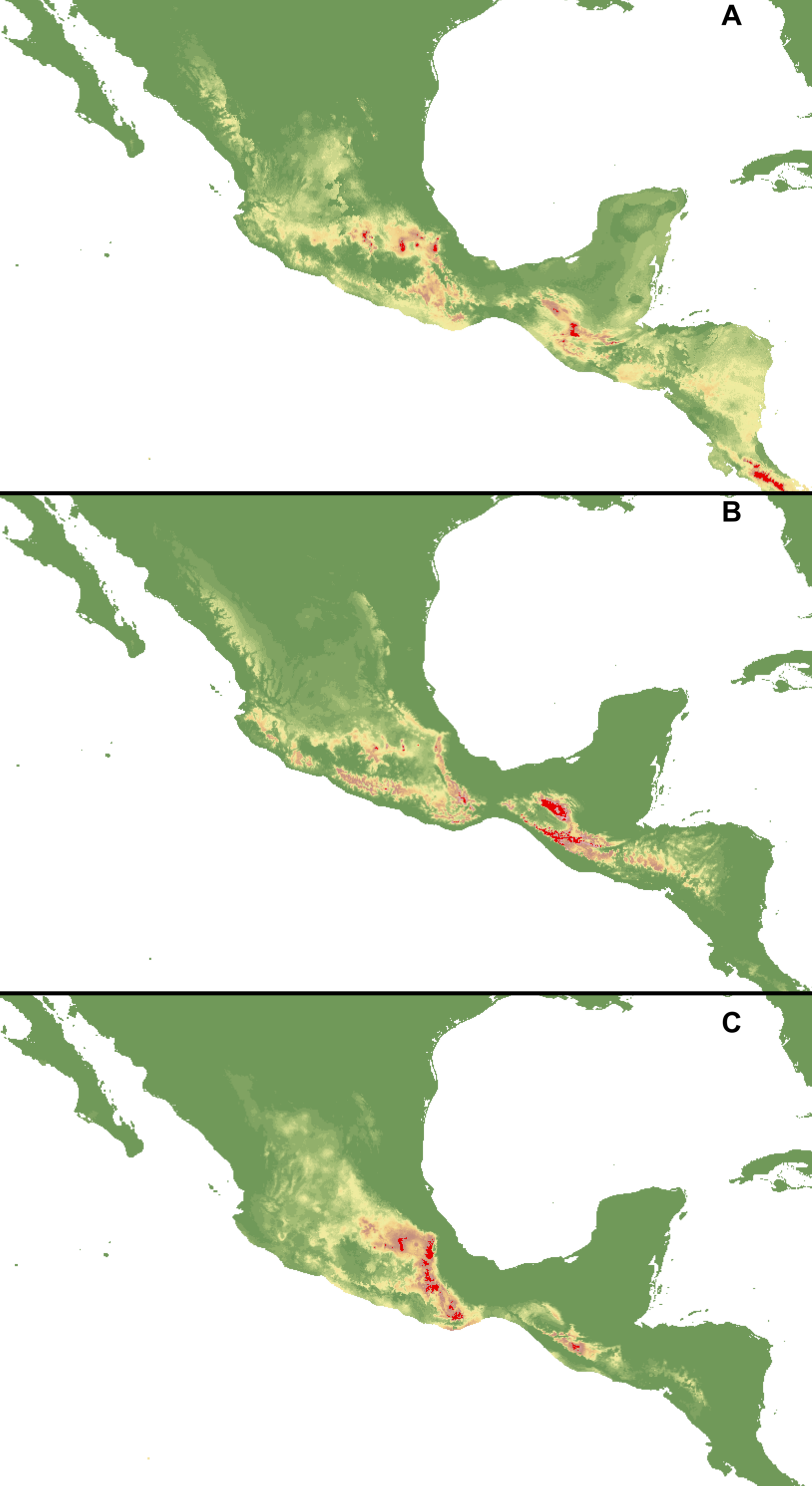
Distribution of suitable habitats of *C. dickinsonianum* (A),* C. irapeanum* (B) and *C. molle* (C) based on the most reliable MaxEnt model. Maps generated in ArcGis 9.2 (http://www.esri.com/).

**Figure 3:**
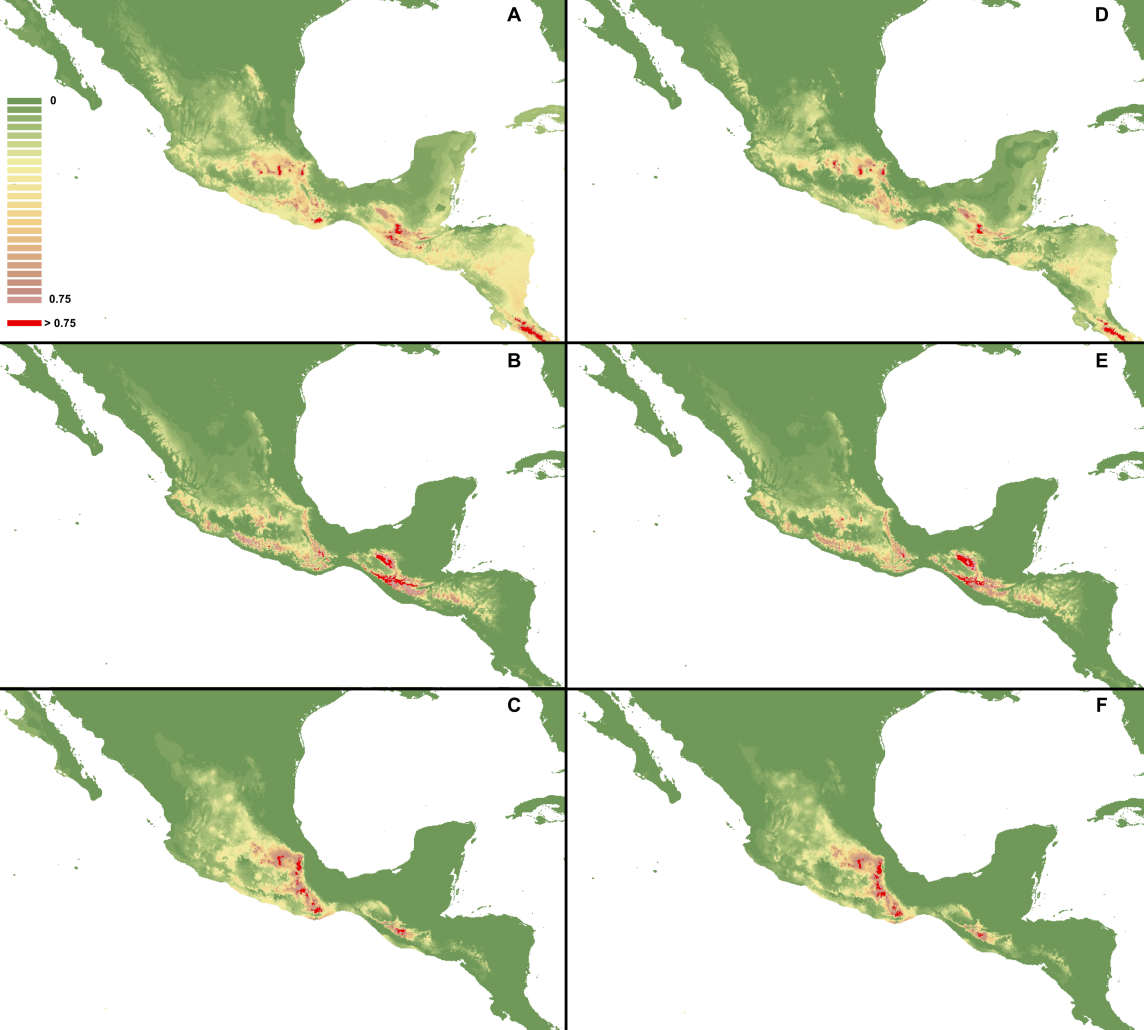
MaxEnt models created based on occurrence data with reduced sample bias. I. Using selected variables: *C. dickinsonianum* (A), *C. irapeanum* (B), *C. molle* (C). II. Using all bioclimatic variables: *C. dickinsonianum* (D), *C. irapeanum* (E), *C. molle* (F). Maps generated in ArcGis 9.2 (http://www.esri.com/).

**Figure 4:**
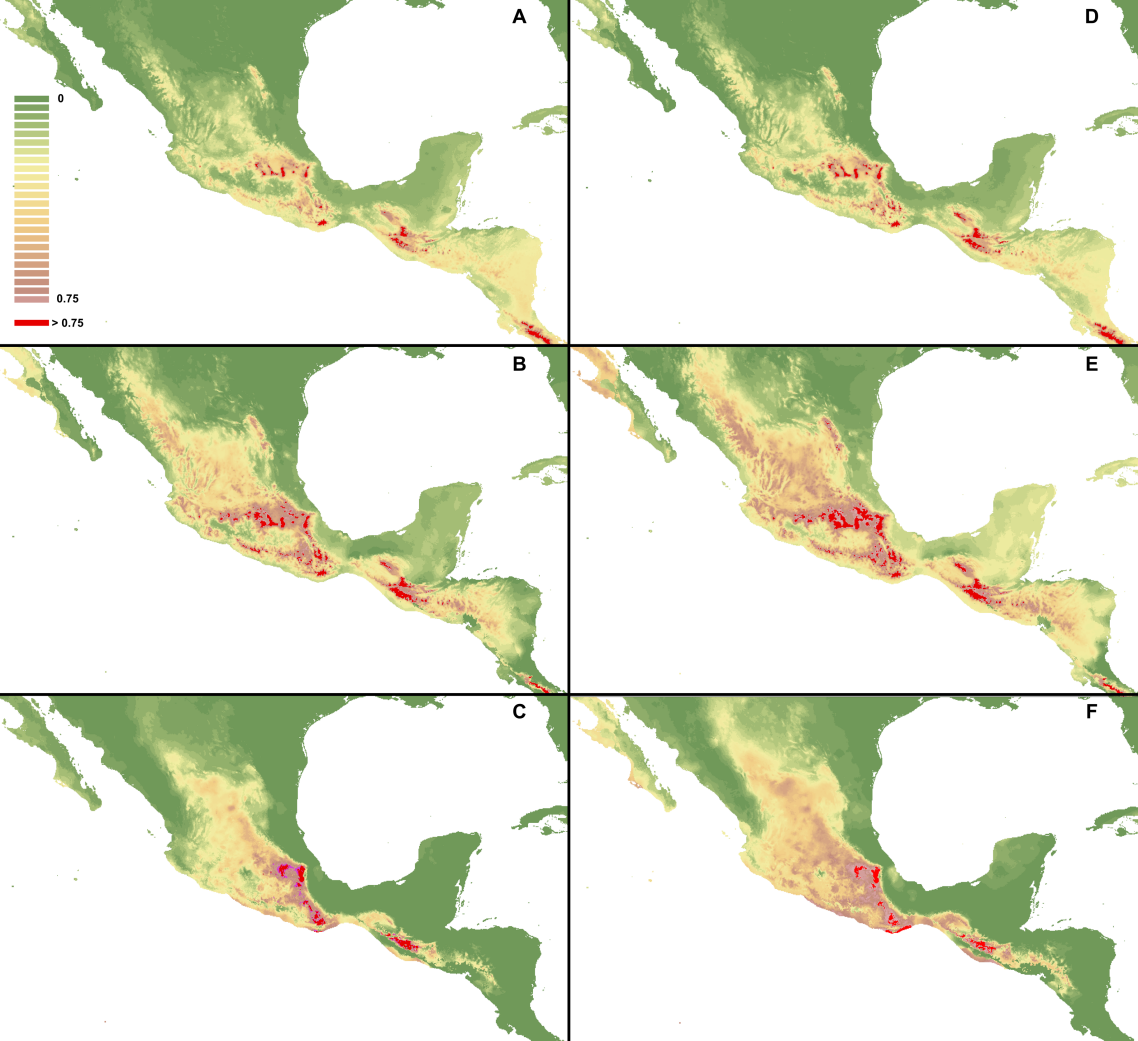
MaxEnt models created based on all gathered occurrence data. I. Using selected variables: *C. dickinsonianum* (A), *C. irapeanum* (B), *C. molle* (C). II. Using all bioclimatic variables: *C. dickinsonianum* (D), *C. irapeanum* (E), *C. molle* (F). Maps generated in ArcGis 9.2 (http://www.esri.com/).

**Figure 5:**
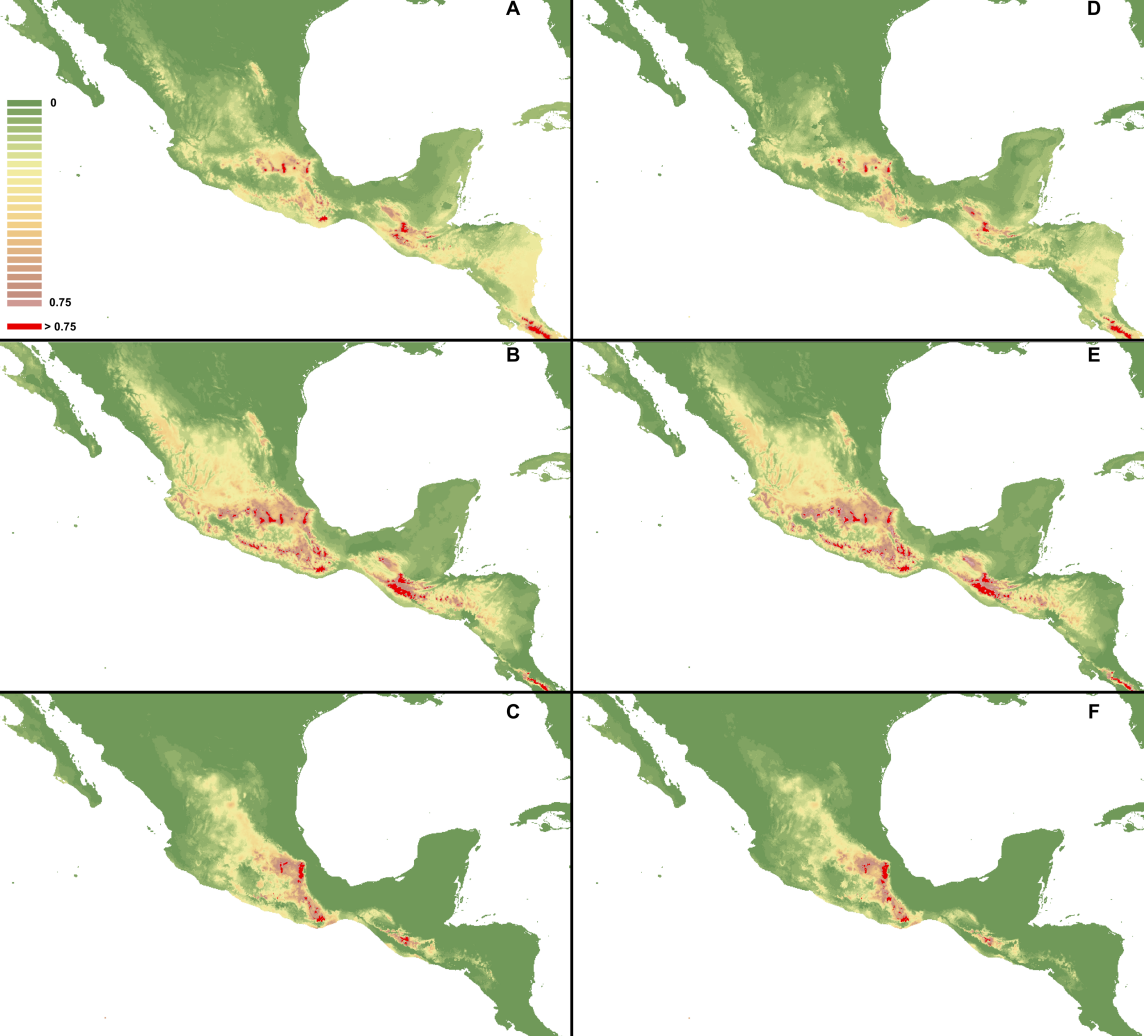
MaxEnt models created with modified regularization multiplier. I Regularization multiplier = 2: *C. dickinsonianum* (A), *C. irapeanum* (B), *C. molle* (C). II. Regularization multiplier = 4: *C. dickinsonianum* (D), *C. irapeanum* (E), *C. molle* (F). Maps generated in ArcGis 9.2 (http://www.esri.com/).

The calculated AUC values for all the created models received high scores of over 0.9 (Tables 3 and 4). Based on this test, the most reliable models were created using all available occurrence and climatic data with default regularization multipliers (1). These are presented in Fig. 2. All other models are provided in Figs. 3–5. According to the most reliable models, the factors limiting the distribution of the three studied species are related to the altitude and temperature (temperature seasonality and mean temperature of the warmest quarter). However, in the models created with a reduced climatic variable dataset some additional factors were indicated as influencing the analysis, e.g., bio2, bio12, bio13, bio1, bio8 and bio19 (Table 5). In addition, their contribution in particular models varied between the species. The niche overlap statistics (Table 6) indicated that the highest probability of co-occurrence between the studied *Cypripedium* species is observed within *C. dickinsonianum* and *C. irapeanum* (*I* = 0.721, *D* = 435) and this was also confirmed in the same statistics calculated for three other datasets (Table 6).

**Table 3:** The average training AUC for replicate runs of various datasets with default regularization multiplier. Standard deviation value given in parenthesis.

	*C. dickinsonianum*		*C. irapeanum*		*C. molle*	
	All localities	Selected localities	All localities	Selected localities	All localities	Selected localities
All variables	0.979 (SD = 0.016)	0.978 (SD = 0.018)	0.977 (SD = 0.017)	0.934 (SD = 0.021)	0.991 (SD = 0.004)	0.988 (SD = 0.005)
Selected variables	0.972 (SD = 0.012)	0.972 (SD = 0.014)	0.971 (SD = 0.038)	0.932 (SD = 0.021)	0.989 (SD = 0.004)	0.985 (SD = 0.006)

**Table 4:** The average training AUC for replicate runs of datasets using various regularization multiplier.

	*C. dickinsonianum*	*C. irapeanum*	*C. molle*
Regularization multiplier = 2	0.965 (SD = 0.013)	0.923 (SD = 0.0.23)	0.985 (SD = 0.007)
Regularization multiplier = 4	0.965 (SD = 0.013)	0.918 (SD = 0.023)	0.977 (SD = 0.015)

The ENM analysis indicated several regions characterized by bioclimatic conditions suitable for the studied species located outside their known geographical ranges (Cribb & Soto-Arenas, 1993). For *C. dickinsonianum*, such areas may be found in the Mexican Volcanic Axis, the Southern Sierra Madre and the Chorotega volcanic front (Fig. 2A). The model of suitable niche distribution created for *C. irapeanum* is quite consistent with its known geographical range (Fig. 2B) with additional potential habitats in the eastern Sierra Madre del Sur. In the Chorotega volcanic front, the area indicated in ENM analysis as suitable for *C. molle* (Fig. 2C), no populations of this species have been found thus far.

The ENM analysis indicated two areas characterized by habitats suitable for all three studied species: the Sierra Madre de Chiapas and the Cordillera Neovolcánica. Within these regions the potentially available habitats for *C. dickinsonianum*, *C. irapeanum* and *C. molle* are separated by less suitable zones. The potential hybrid zones of *C. irapeanum* and *C. molle* are located in the eastern Sierra Madre del Sur.

### Molecular analysis

**Figure 6:**
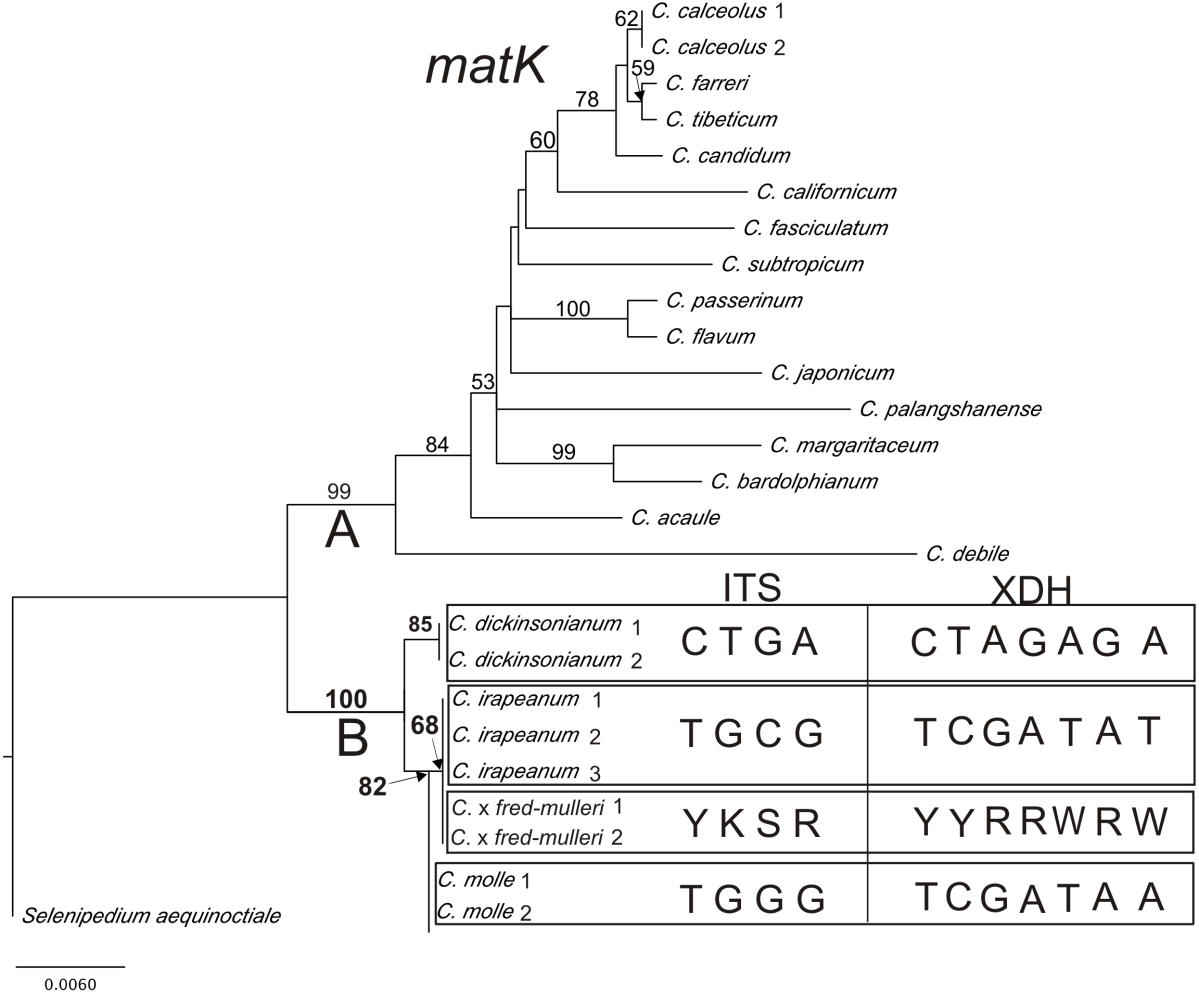
The phylogenetic tree based on *matK* gene sequences obtained by the maximum-likelihood method for *Cypripedium*. Bootstrap percentages (BP) >50 are given for supported clades above branches. Polymorphic sites in the alignment of ITS and *Xdh* for *C. dickinsonianum, C. irapeanum, C. molle* and *C.* × *fred-mulleri* are given.****

Results from phylogenetic analyses based on the plastid *matK* gene are presented in a phylogram (Fig. 6). Bootstrap support (BS) above 50% is given for supported clades above branches. The *matK* tree can be divided into two highly supported clades (A = 99 and B = 100). Clade A consists of species represented by various sections of *Cypripedium*. The base of the tree (clade B) comprises three species from section *Irapeana. C. irapeanum* together with the putative hybrid composing one clade which is a sister to *C. molle*. *C. dickinsonianum* is a sister to them. Pairwise alignment of nuclear ITS, *Xdh* and the plastid sequence comprising the 5′end of the intron *trnK* and *matK* gene revealed significant differences between *Cypripedium irapeanum* and *C*. *dickinsonianum*. Within the ITS sequence, four substitutions were observed—two transversions and two transitions. In addition to the sequence of ITS2, an indel of 15 base pairs in length occurred. Sequences (chromatograms) of putative hybrids are noisy (weak) from that site (sequences from two different alleles overlap each other making chromatograms unreadable). This feature was observed in both forward and reverse strands (see the chromatogram file provided as Supplemental Information). Within the *Xdh* sequence, seven substitutions were observed, of which five were transitions. Two specimens of *Cypripedium* which exhibited characteristics of hybrids in polymorphic sites have double peaks corresponding to nucleotides found in both species (Table 7). Comparison of the plastid sequence between *C. irapeanum* and *C*. *dickinsonianum* showed an indel of seven base pairs in length at the 3′*trnK* intron and five transitions and one indel in the *matK* gene. *C. irapeanum* and the putative hybrid have identical sequences of the *matK* gene. Comparison of molecular markers identified three substitutions between *C. irapeanum* and *C. molle*, one in each of the analyzed markers. DNA data matrices are provided as Supplemental Information.

### Taxonomic treatment

Due to the detection of gene flow between *C. dickinsonianum* and *C. irapeanum* and mixed morphological characters of the population discovered by Mr Muller in Guatemala we decided to describe it as the first, natural hybrid in the section *Irapeana* under the name *Cypripedium* × *fred-mulleri*.

*Cypripedium* × *fred-mulleri* Szlach., Kolan. & Górniak, *hybr. nov.*

Diagnosis: *Cypripedium* × *fred-mulleri* is characterized by having flowers 5.2–7 cm across, elliptic, acute dorsal sepal, oblong-elliptic, obtuse petals, deeply saccate, obovoid-globose lip and trullate, acute staminode. It differs from *C. irapeanum* in its smaller flowers, deeper color (closer to *C. dickinsonianum*), density of windows on the lip, and form of dorsal sepal and petal apex. From *C. dickinsonianum* it is distinguished, inter alia, by the shape of the staminode and lip as well as by the petal form.

Type: Guatemala, Alta Verapaz. South of Cobán. 30 May 2013. *F. Muller s.n.* (BIGU! 309 holotype). UGDA-DLSz! - drawing of type, photos.

**Figure 7:**
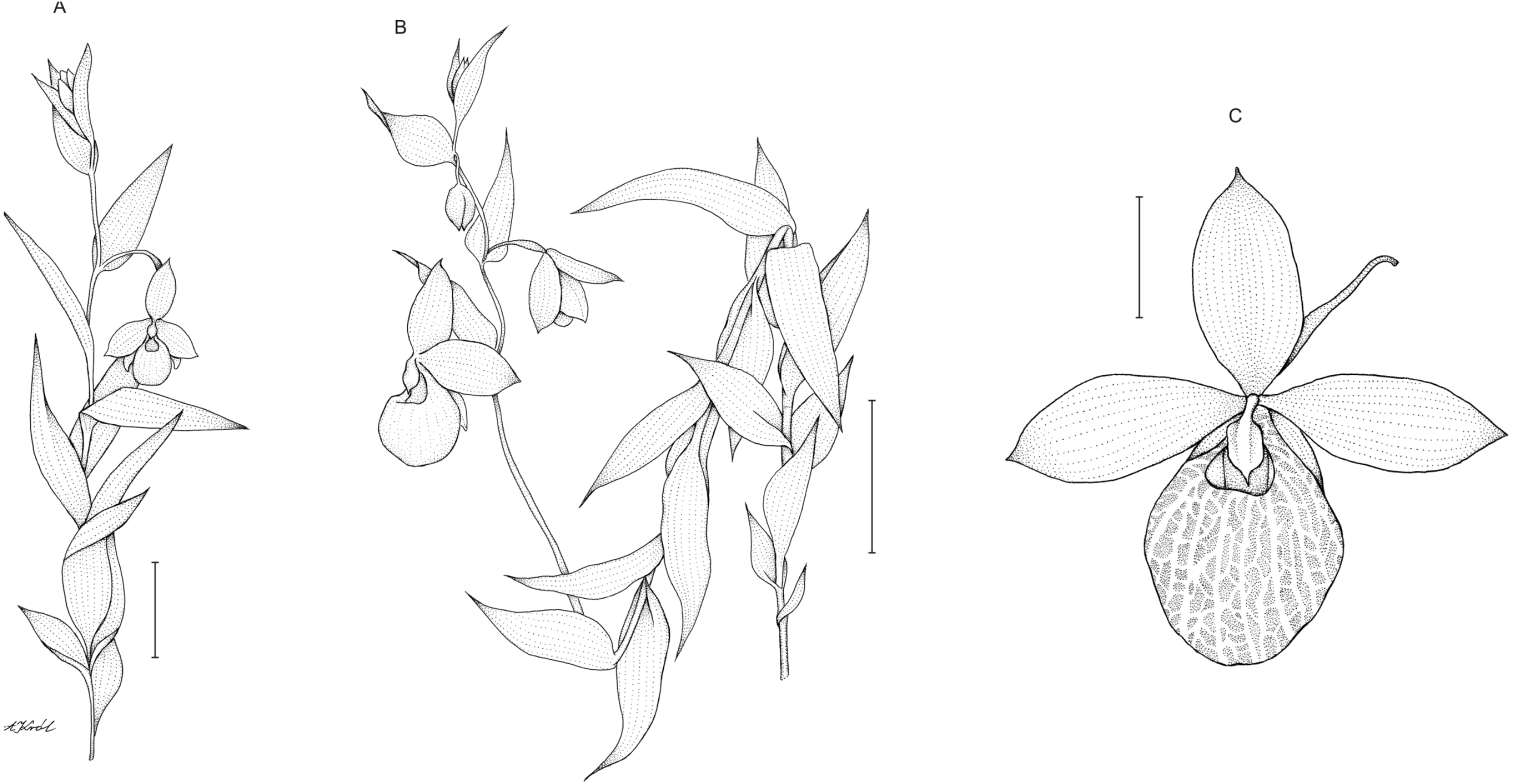
*Cypripedium*×*fred-mulleri*. Habit (A–B). Scale bars = 5 cm. C –flower (C). Scale bar = 2 cm. Drawn by A Król.

Description: Plants up to 75 cm tall, densely and softly hairy throughout. Stem erect, rather stout. Leaves up to 15 cm, distributed along the stem, 3–8 cm long, 2.8–3.8 cm wide, ovate to ovate-lanceolate, acute. Inflorescence 15–33 cm long, loosely 5–8-flowered. Flowers showy, large, yellow. Floral bracts 4–6.6 cm long, ovate-lanceolate, acute. Pedicel up to 1 cm long, pubescent. Ovary up to 2.5 cm long, pubescent. Dorsal sepal 3–3.8 cm long, 1.7–2 cm wide, elliptic, acute, margins pilose. Petals 3.4–4.3 cm long, 1.6–2.1 cm wide, oblong-elliptic, obtuse, pilose, especially near the base. Synsepal 2.2–3.2 cm long, 1.6–2 cm wide, elliptic, obtuse, bifid or occasionally free to the base, margins pilose. Lip 3.5–4 cm long, 2.8–3 cm wide, deeply saccate, obovoid-globose, margins incurved around the lip opening, with translucent windows all over the surface. Staminode 1.2 cm long, 0.7–0.8 cm wide, trullate, acute. Capsule 2.2–2.6 cm long. Figure 7.

**Table 5:** Relative contributions of the most important environmental variables to the Maxent models created with various datasets.

	*Cypripedium dickinsonianum*		*Cypripedium irapeanum*		*Cypripedium molle*	
	All localities	Selected localities	All localities	Selected localities	All localities	Selected localities
All variables	Bio4 (33.3)	Bio4 (41.3)	Bio4 (33)	Alt (30.7)	Bio4 (27.8)	Alt (27)
	Bio10 (16.4)	Bio10 (23.9)	Bio10(16.1)	Bio4 (24.5)	Alt (27.2)	Bio4 (24.6)
	Alt (16.4)	Alt (12.4)	Alt (13.3)	Bio10 (23)	Bio10 (9.8)	Bio19 (16.4)
Selected variables	Bio2 (22.6)	Bio4 (48.3)	Bio2 (22.6)	Alt (39.1)	Bio2 (22.6)	Alt (29.5)
	Bio12 (21.2)	Alt (22.6)	Bio12 (21.2)	Bio4 (26.9)	Bio12 (21.2)	Bio4 (24.4)
	Bio13 (18.4)	Bio1 (8.1)	Bio13 (18.4)	Bio8 (18.8)	Bio13 (18.4)	Bio19 (23.8)

**Table 6:** Niche overlap between *C. dickinsonianum*, *C. irapeanum* and *C. molle*. (1–3)—models based on all available occurrence data and bioclimatic variables dataset; (4–6)—models based on all available occurrence data and selected bioclimatic variables dataset; (7–9)–models based on selected occurrence data and all available bioclimatic variables; (10–12)–models based on selected occurrence data and reduced dataset of bioclimatic variables.

	D∖I	*C. dickinsonianum*	*C. irapeanum*	*C. molle*
1.	*C. dickinsonianum*	x	0.721	0.614
2.	*C. irapeanum*	0.435	x	0.659
3.	*C. molle*	0.364	0.391	x
4.	*C. dickinsonianum*	x	0.713	0.616
5.	*C. irapeanum*	0.424	x	0.671
6.	*C. molle*	0.354	0.409	x
7.	*C. dickinsonianum*	x	0.870	0.583
8.	*C. irapeanum*	0.633	x	0.734
9.	*C. molle*	0.341	0.479	x
10.	*C. dickinsonianum*	x	0.909	0.609
11.	*C. irapeanum*	0.695	x	0.733
12.	*C. molle*	0.358	0.483	x

Paratypes: Guatemala. Alta Verapaz, South of Cobán. 25 Jun 2009. (Muller - photo!); The same location 26 Jun 2010. (Muller - photo!).

Etymology: Dedicated to the discoverer of this hybrid, Fred Muller.

**Figure 8:**
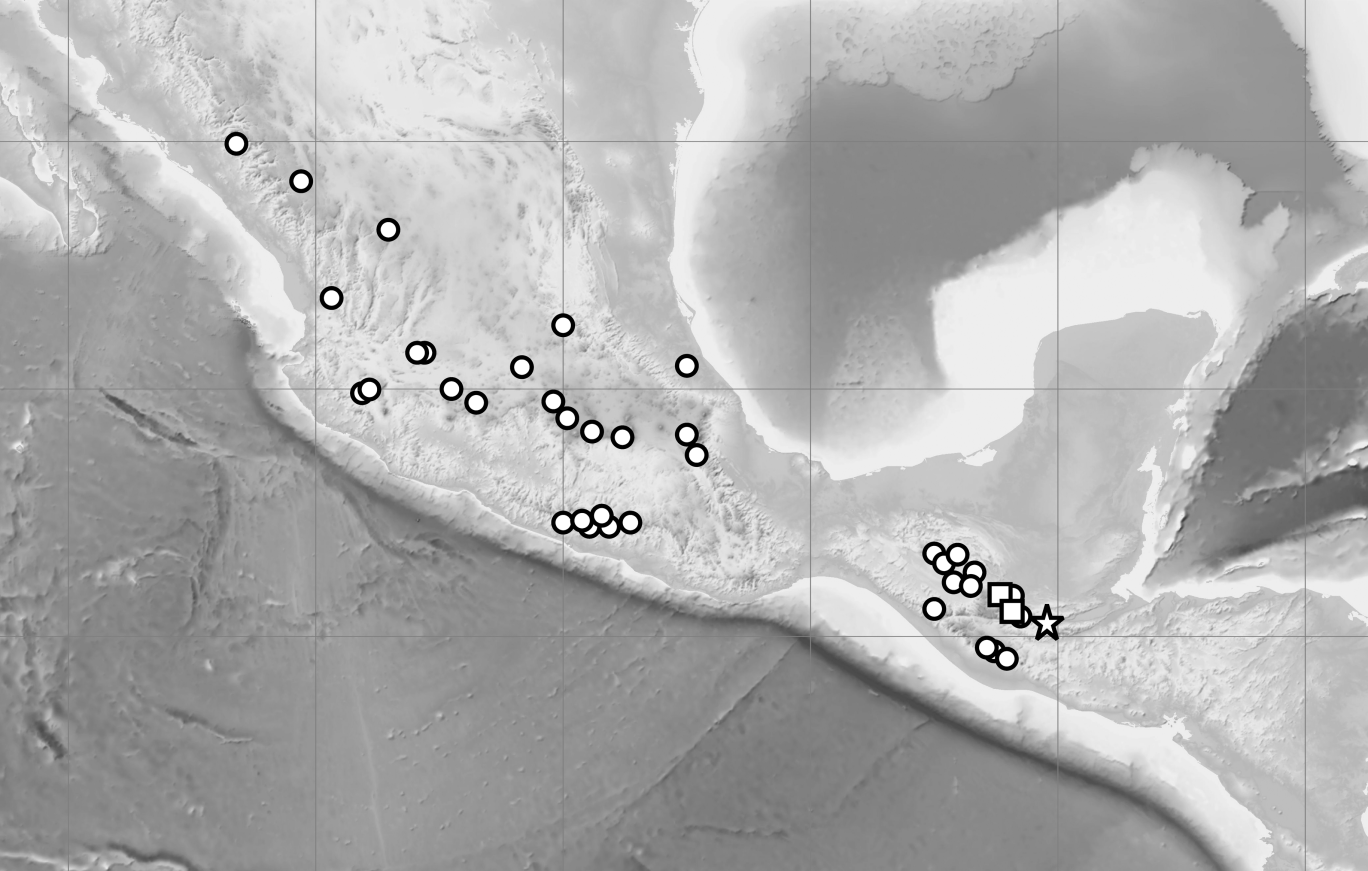
Distribution of *C. irapeanum* (spot), *C. dickinsonianum* (square) and *C.*×*fred-mulleri* (star). Cribb & Soto-Arenas (1993), modified. Map generated in QGIS 2.2.0 (QGIS Development Team, 2016).

Notes: Morphologically, *C.* × *fred-mulleri* is transitional between its parental species in many respects, as we describe in Table 1. The hybrid occupies an intermediate position in the general size of the plant, number of leaves and inflorescence length. For example, according to literature data and our own study, the inflorescence of *C. irapeanum* reaches up to 40 cm in length, whereas in *C. dickinsonianum* it is less than 9 cm. The length of inflorescence of *C.* × *fred-mulleri* is between 15 and 33 cm. This inflorescence can bear five to eight flowers. The reported number of flowers per inflorescence in *C. irapeanum* is up to 12, and in *C. dickinsonianum* it is between two and six (Figs. 9 and 10). Even the number, size and distribution pattern of diaphragmatic windows on the lip is manifestly transitional between both parental species. In *C. dickinsonianum* the diaphragma is outspread between somewhat thickened, dendritic veins and cover ca 40% of the total lip surface. On the other hand, in *C. irapeanum* the windows are relatively small and occupy less than 10% of the lip surface. The lip of *C.* × *fred-mulleri*, although in form similar to the ovule parent, is covered by diaphragma in a similar pattern as in its pollen parent which cover ca 30% of whole lip surface. In some respects, however, *C.* × *fred-mulleri* is more similar to its pollen parent (densely hairy stem and leaves, length of the leaf blade), but in some others to its ovule parent. This set of characteristics concern the form and width of the leaf blade, general flower architecture, and the length and general form of staminode. It is noteworthy that in *C.* × *fred-mulleri* all taxonomically important characters useful in determination of Neotropical *Cypripedium* species, i.e., number of flowers per inflorescence, size of the flower segments and generative parts, are intermediate between parental species.

Distribution: Known so far to be exclusively from the Guatemalan department of Alta Verapaz. Due to the vulnerability of populations of *C. irapeanum, C. dickinsonianum* and *C.* × *fred-mulleri* to illicit harvesting, the exact locality is not given. The known localities of *C. irapeanum* are distributed from Central Mexico to Guatemala and Honduras while the currently known range of *C. dickinsonianum* is discontinuous, extending from eastern Chiapas (México), through the Sierra de los Cuchumatanes and the Sierra de Chamá to the central Honduran uplands (although herbarium vouchers are currently lacking Dix & Dix, 2000). Figure 8.

Ecology: The hybrid population was found on a south-oriented limestone hillside at an altitude of about 1,500 m. The plants grow in an open, seasonally dry pine-oak forest with *Brahea dulcis* (Kunth) Mart. (Arecaceae) and species of *Agave* L. (Asparagaceae). Other terrestrial orchid species occurring in this area are: *Cyrtopodium punctatum* (L.) Lindl., *Stenorrhynchos pubens* (A. Rich. & Galeotti) Schltr*.* and *Dichromanthus cinnabarinus* (La Llave & Lex.) Garay. Moreover, two species of *Bletia* Ruiz & Pav. have been reported from this location. The hybrid plants begin blooming in mid-May, at the beginning of the rainy season. The flowers have been observed as late as at the end of July, which is the beginning of the flowering season for both *C. irapeanum* and *C. dickinsonianum* in nearby colonies. Field observations in 2013 suggested that the population might have benefited from a recent wild fire, as a significant increase in the number of flowering specimens had previously been recorded in the season following a fire at the locality.

**Table 7:** Polymorphic sites in the alignment of ITS1-5.8S-ITS2, Xdh and 3′trnK-matK sequences. “–”, indicate indel in the alignment; “+”, indicate base pair in the alignment; Y, C and T; K, G and T; S, C and G; R, A and G; W, A and T.

Base position in the matrix																			
	ITS1-5.8S-ITS2					*XDH*							3′ *trn* K*- mat*K						
	8	99	208	587	629–643	9	18	176	217	672	684	779	15–21	92	299–304	318	511	817	1171
*C. dickinsonianum*	C	T	G	A	+	C	T	A	G	A	G	A	–	C	–	A	G	A	C
*C. irapeanum*	T	G	C	G	–	T	C	G	A	T	A	T	+	T	+	G	A	G	T
*C*. ×*fred-mulleri*	Y	K	S	R	noisy (weak)	Y	Y	R	R	W	R	W	+	T	+	G	A	G	T
*C. molle*	T	G	G	G	–	T	C	G	A	T	A	A	+	T	+	G	A	A	T

### Key to the taxa of *Cypripedium* sect. *Irapeana*

**Table:** 

1.	Staminode suborbicular, shortly apiculate	*Cypripedium molle* Lindl.
1.	Staminode trullate to cordiform or transversely elliptic, acute to apiculate	2
2.	Lip small, less than 3 cm long	*Cypripedium dickinsonianum* Hágsater
2.	Lip large, over 3.5 cm long	3
3.	Inflorescence less than 6-flowered, less than 25 cm	*Cypripedium irapeanum* La Llave & Lex.
3.	Inflorescence 5-8-flowered, 15–33 cm long	*Cypripedium × fred-mulleri* Szlach. et al.

### Ovule and seed development

The seeds inside four open-pollinated flowers did not react with TTC as was indicated under stereomicroscope (Figs. 11A–11E). Deeper analysis revealed that the enlarged capsules contained a mix of unfertilized (Figs. 12A–12G) or embryo-bearing ovules (9.2–26.2%; Fig. S1). In the two ovaries, a small number of embryo-bearing ovules was accompanied by many unfertilized ovules that were at maturity (Figs. 12F–12G) or aborted (Fig. 11E). Very early stages (from the zygote to a few-celled proembryo) of embryo development were detected inside ovules/young seeds (Figs. 11E, 12H–12J). The ovaries without embryos contained ovules that were mostly at the bisporic stage (Figs. 12B–12C), sporadically at modified monosporic megasporogenesis stages (Fig. 12D), or at megagametogenesis (Fig. 12E).

**Figure 9:**
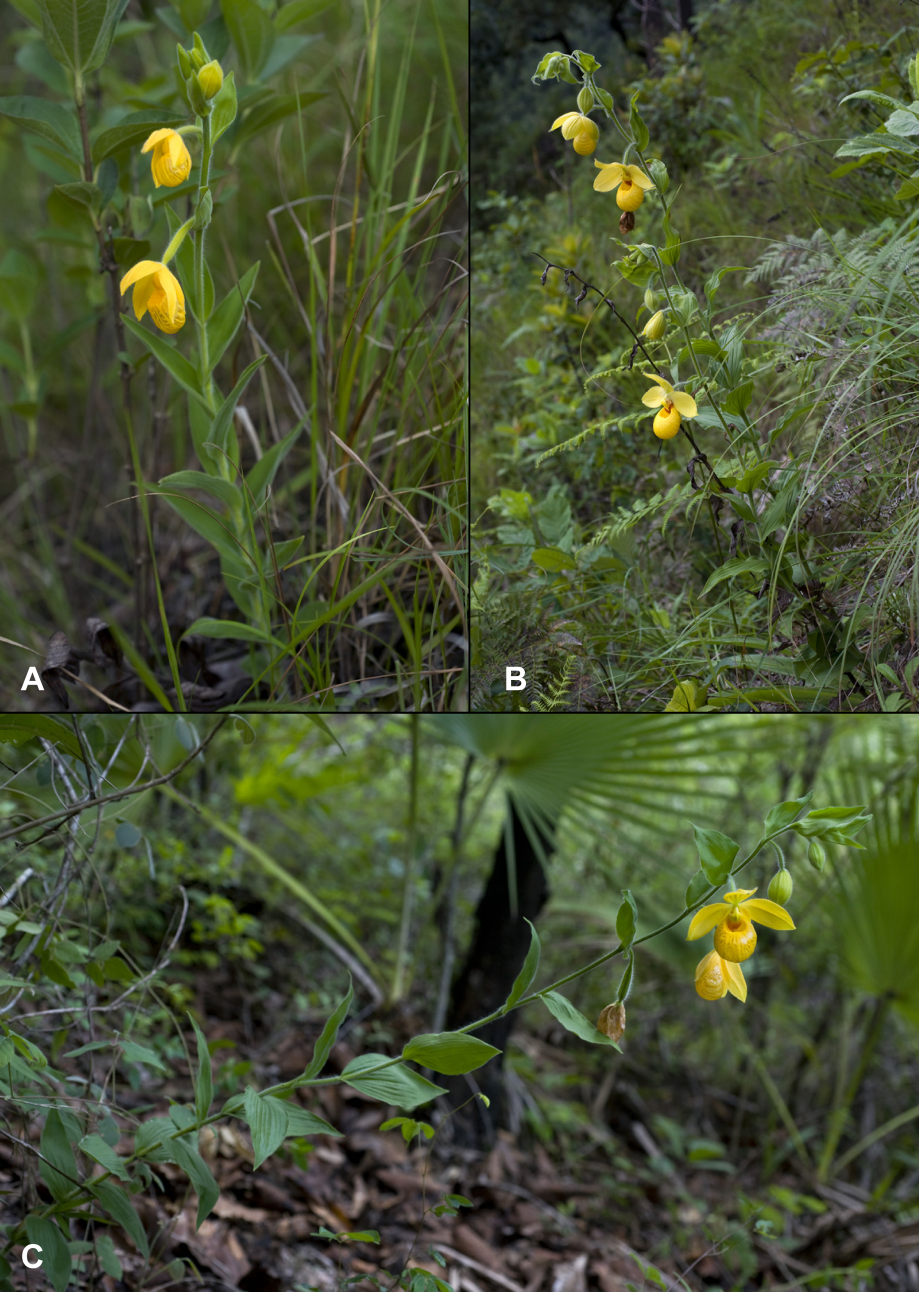
Comparison of the habit of *Cypripedium dickinsonianum* (A), *C. irapeanum* (B) and *C.*×*fred-mulleri* (C). Photos by F Muller.

**Figure 10:**
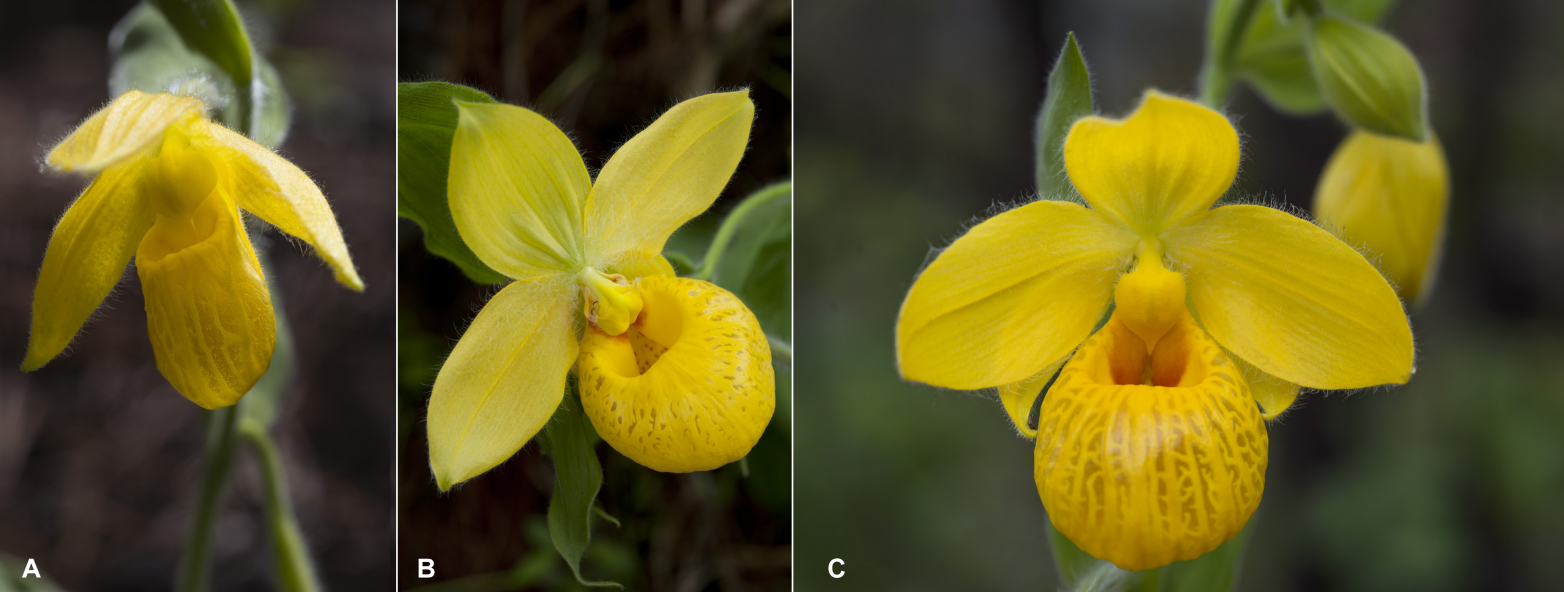
Flowers of *Cypripedium*. *Cypripedium dickinsonianum* (A), *C. irapeanum* (B) and *C*. × *fred-mulleri* (C). Photos by F Muller.

**Figure 11:**
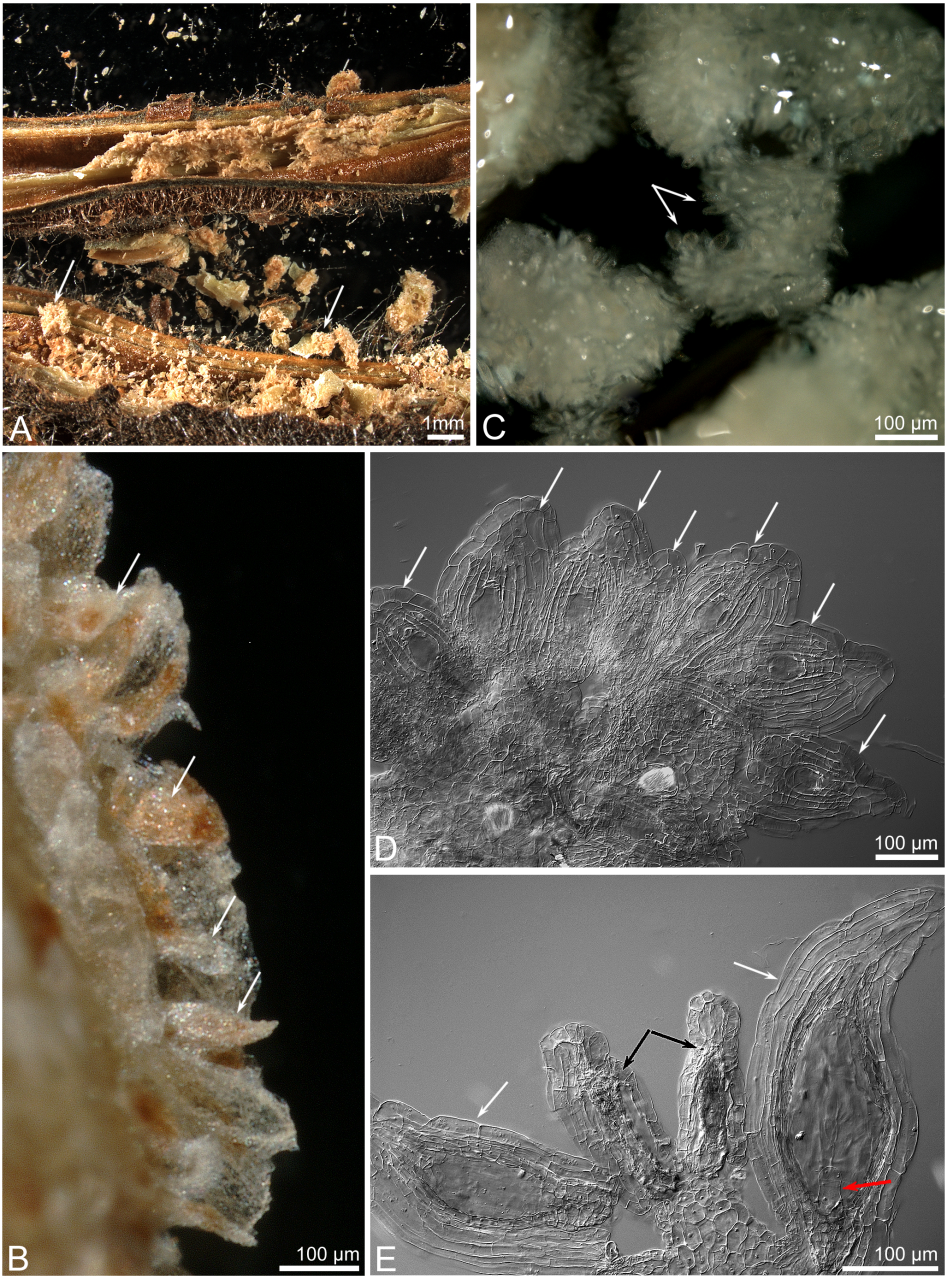
Seed capsule produced in *C.*×*fred-mulleri*. Non-crumbling mass of ovules/ seeds (arrows) inside dried capsule (A–B), masses of the ovules after TTC staining (C–D), unfertilized ovules (arrows) at gametogenesis stages (C), and ovules collapsed (black arrows) and enlarged (white arrows), and with embryo (red arrow) (D).

**Figure 12:**
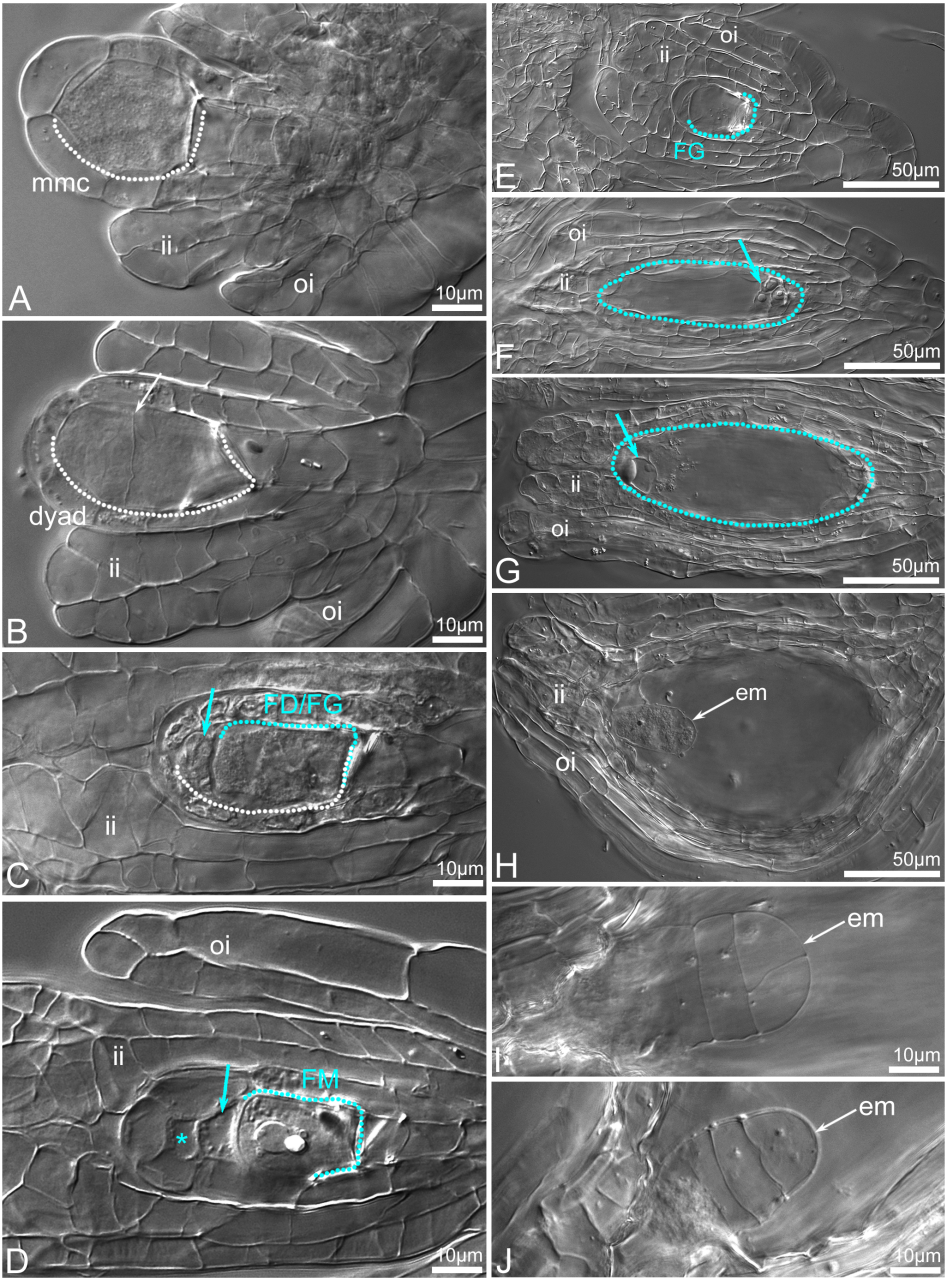
Ovule development before (A–G) and after fertilization (H–J) in *C.*×*fred-mulleri*. Megasporogenesis stages (A–D), megagametogenesis stages (E–G), embryogenesis stages (H–J). The MMC in young ovules. The inner integument has been initiated and grows towards the megasporocyte, and the outer integument begins to appear (A). At dyad stage, the chalazal cell of the dyad is larger than the micropylar cell (the boundary between dyad cells is indicated by arrow) (B). At binucleate dyad stage, the micropylar cell of the dyad is degenerated (blue arrow) (C). Chalazal cell of the dyad (FD) is enlarged, binucleate and initiates 2-nucleate FG development. At dyad or triad stage, a viable chalazal megaspore-like cell (FM) assisted by small and degenerated cell (arrow). The origin of the top micropylar cell could be meiotic or nucellar (star). The inner and outer integuments are developmentally advanced; the inner integument encloses the nucellus (D). A young (two- or four-nucleate) FG stage; the inner integument encloses the nucellus (E). At maturity (F–G), a few cells of gametophyte (arrows) are visible at chalazal (F) and micropylar (G) pole of FG. The inner integument is adhered to the embryo sac. At 2-/3-celled stage of embryo development. Both the inner and outer integuments have completely covered the embryo sac forming seed coat (H). A T-shape proembryo (I). A few-celled proembryo (J). Abbreviation: arch, archespore cell; ii, inner integument; oi, outer integument; mmc, megaspore mother cell; FD, functional dyad; FM, functional megaspore; em, embryo. The MMC and following cells are outlined by a dashed white line. The FM and FG are outlined by a dashed blue line. Clearing material visualized by DIC (differential interference contrast microscopy).

## Discussion

Interspecific hybridization seems to be an important factor in the process of evolution of angiosperms. It appears to be a common phenomenon in Orchidaceae (Pinheiro et al., 2010; Moraes et al., 2013; Marques et al., 2014). Many species arise from both homoploid and heteroploid hybridization. A homoploid hybrid species has the same ploidy level as its progenitors and tends to have a combination of alleles that are specific to either parents (Rieseberg, 1997). Natural hybridization of *Cypripedium* species has been reported only a few times, despite the relatively large number of species recognized in the genus (37—Eccarius, 2009; 45—Cribb, 1997), the huge area of geographical distribution across the northern hemisphere and the numerous ecosystems inhabited by these orchids. In theory, many species have the potential to hybridize as many of them are known to be sympatric. Cribb (1997) listed only four natural hybrids in the genus, whereas Eccarius (2009) provided additional information bringing the total to ca. 10, eight of which have been formally described while two remain undescribed. Amongst these natural hybrids, Eccarius (2009) mentioned a putative natural hybrid between the Neotropical *C. irapeanum* and *C. dickinsonianum* based on Guatemalan material obtained from Fred Muller in 2008.

For taxonomic studies and hybrid identification, amplified fragment length polymorphism markers (AFLP), nuclear single simple repeat (SSR) analysis have been widely used (respectively Marques et al., 2014; Pinheiro et al., 2010) to determine genetic structures of hybrid zones. In our case basic Sanger sequencing proved the hybrid origin of the putative hybrid. Specimens with intermediate flowers between *C*. *irapeanum* and *C*. *dickinsonianum* within the ITS and *Xdh* (both nuclear markers) sequences have the signal sequence of both the above species (Table 7, Fig. 6). The analysis of plastid sequences indicated that the maternal line is *C*. *irapeanum*. The latter species and *C. × fred-mulleri* have identical plastid (*matK*) sequences. Our data indicate that some portion of the genome (at least one or two chromosomes) of *C*. *dickinsonianum* flow to the gene pool of *C*. *irapeanum*. Molecular analyses confirmed the hybrid origin of the plants discovered by Mr Muller. The morphological data do not strictly confirm the hybrid origin of the plants as, in terms of floral morphology, *C*. × *fred*-*mulleri* is more similar to *C*. *irapeanum* than to *C*. *dickinsonianum*. Thus, hybrid species do not always have intermediate characters. Rieseberg (1995) even stated that one of the most common misconceptions is that hybrids are typically morphologically intermediate between their parents. Several authors (e.g., Bateman & Farrington, 1987; Bateman & Hollingsworth, 2004; Bateman, Smith & Fay, 2008; R Bateman, pers. comm., 2017) indicated a strong asymmetry of phenotypically expressed inheritance of orchid hybrids relative to their parent. What is interesting is that all hybrid species from the above articles resembled their seed parent. One of the possible explanations of this phenomenon could be the influence of the cytoplasm on nuclear gene expression (Bateman, Smith & Fay, 2008). Secondly, multiple introgression into one parental line may bring hybrid generations reminiscent of this one parent (e.g., Pinheiro et al., 2010; Schilling, 2016 and references cited therein). Based on this information and our molecular data we think that similar morphology of the flowers of *C*. *irapeanum* and *C.* × *fred-mulleri* is not an argument against the hybrid origin of the latter. Future study should include more samples for molecular analyses to confirm if there is gene flow between hybrid individuals. In that case, we should observe both homozygotes and heterozygotes in the F2 generation. The second aim of any future study should be the detection of whether *C. dickinsonianum* is the seed parent and a determination of the degree and direction of the introgression of the nuclear genome of both species into the hybrid population. However, based on visual inspection in the field, the putative hybrid grew only within a *C*. *irapeanum* population. This additionally supports this species as being the seed parent and confirms our molecular data. Identification of natural hybrids and the observation of several successive generations can be a valuable source of information on how to overcome the barriers between species. There are several possible scenarios for the further evolution of these hybrids. A new ecological niche would separate them from the parent species preventing gene flow/introgression. Alternatively, remaining in the niche of the parent species can lead to the elimination of less-adapted hybrids and/or introgression of genes into the genome of the hybrids, which will result in an increase of genetic diversity of parental lines. Hybrid populations, especially the F1 generation, are burdened by a reduction of fertility resulting in both poor seed viability and production of unbalanced gametes (Rieseberg, 1997). Observations made by Fred Muller—at a Guatemalan locality where *C. irapeanum* and *C. dickinsonianum* occur in close sympatry—showed that both species are pollinated by small *Trigona* Jurine species as well as other genera of small bees (including unidentified sweat bees—family Halictidae). A high percentage of fruit set was noted for this population. The enlarged ovaries of our putative *C.* × *fred-mulleri* hybrid contained seeds without embryos or 9.2–26.2% of seeds with embryos, in contrast to a high number (73.8–100%) of ovules which were unfertilized or aborted (Fig. S1). Despite the embryos being too young (few-celled) to be detected via a TTC test (see Lee et al., 2005 for details of *C. formosanum* seed viability, ranged from 27.4 to 47.4%), they might develop further. Even in such cases, the efficiency of seed production was difficult to estimate, because an ovary contains thousands to millions of ovules, with that number decreasing during around-pollination and post-pollination events (Cress, 1981; Nazarov & Gerlach, 1997). Finally, the TTC test commonly counts the embryo-bearing ovules (seeds) but does not include all of the ovules (i.e., fertilized and unfertilized) inside the ovary (for details of TTC use, see Lee et al., 2005; Zeng et al., 2014). The fertilization of only around 25% of the fertilizable *C. × fred-mulleri* ovules may indicate the presence of some late post-mating barrier between *C. irapeanum* and *C. dickinsonianum*. This phenomenon is very common in other food-deceptive orchid species (Cozzolino & Scopece, 2008). Hybridization, regarded as a main inducer of largely sterile hybrids, can provide important explanation of mechanisms that prevent introgression and, consequently, maintain parental species integrity (Pinheiro et al., 2010). All postzygotic isolation stages generally evolved gradually over time and late-acting postzygotic barriers, such as hybrid sterility and hybrid inviability, evolved faster than embryo mortality (Scopece, Widmer & Cozzolino, 2008). On the other hand, the indication of non-disturbed development of ovules and megagametophytes makes the *C.* × *fred-mulleri* hybrid most likely fertile. Our finding of a bisporic type of megasporogenesis is congruent with sporogenesis in other *Cypripedium* species (Carlson, 1945; Sood & Mohana Rao, 1988; Vinogradova & Andronova, 2002; Yeung & Low, 1997 and references cited therein). In addition, we discovered a triad of megaspores in some ovules, indicating a modified monosporic pathway and showing the possibility of (at least) two modes of embryo sac formation in *C.* × *fred-mulleri*, as in *Microstylis musifera* (Sood & Mohana Rao, 1989), *Malaxis saprophyta* (Sood, 1992) and in other examples of intraspecific co-existence of different types of embryo sac development (Vij & Sharma, 1986 according to Yeung & Low, 1997). Thus, the pollination of all four *C.* × *fred-mulleri* ovaries might be possible as all ovaries had been enlarged and ovule developmental events had progressed. A small number of the counted embryos could cause by early stages of seed capsule development (at fertilization and embryogenesis stages) (see Sood & Mohana Rao, 1988; Lee et al., 2005; Zeng et al., 2014 for summary of embryogenesis time table in *Cypripedium*). The enlargement of ovaries, which we also noted in the tested plants, can take place due to successful pollination and sometimes in emasculated flowers and flowers isolated from pollination. Hence, the enlargement of the ovary without pollination may be indicative of a programmed phenomenon (Krawczyk et al., 2016). The genesis and fate of observed *C.* × *fred-mulleri* young seed capsules and embryos remains to be determined, together with consideration of the environmental factors (e.g., pollination limitations) and mechanisms which decrease seed formation efficiency.

The question remains as to the true taxonomic status of the third species of the section *Irapeana*, *C. molle*, which in numerous morphological characters appears to be an intermediate between *C. irapeanum* and *C. dickinsonianum*. It was described by Lindley in 1840 based on Hartweg’s collection from the Mexican city of San Miguel Sola (Oaxaca). Thus far its documented populations are located exclusively in Puebla and Oaxaca States (Cribb & Soto-Arenas, 1993). As reported by Cribb & Soto-Arenas (1993), this species is cross-pollinated and its flowers are visited by small halicid bees bearing pollen. Eccarius (2009) considered *C. molle* to be a subspecies of *C. irapeanum*. In our opinion, morphological differences, especially the form of the staminode and its somewhat disjunct distribution are sufficient reason to continue to treat these two taxa as separate species. *Cypripedium molle* is distinguished from *C.* × *fred-mulleri* by a series of unique morphological characters, such as the form of the staminode (trullate, acute vs. suborbicular, apiculate) and lip (obovoid-globose vs. obovoid), as well as other quantitative features, e.g., flower size, inflorescence length, number of flowers and length of the floral bracts (Table 1).

Based on the available literature information (e.g., Cribb & Soto-Arenas, 1993) and studied herbarium material, there are just two regions where more than one representative of *Cypripedium* sect. *Irapeana* has been found. These are located in the Maya Highlands (*C. dickinsonianum* and *C. irapeanum*) and the eastern part of the Southern Sierra Madre (*C. molle* and *C. irapeanum*). Both these regions were also indicated in ENM analysis as areas of the potential hybridization of the studied species. Additional suitable habitats for all three *Cypripedium* species could be located in the Cordillera Neovolcánica according to the obtained models; however, it should be noted that this region is quite distant from the edges of the known geographical range of *C. molle.*

Previous research has indicated that Maxent can somewhat compensate for incomplete, small species occurrence data sets and produce near maximal accuracy levels in these conditions (Hernandez et al., 2006). However, we believe that in our study the model of *C. dickinsonianum* is overfitted, despite the high AUC calculated for this analysis. While distribution of the suitable habitats of *C. irapeanum* and *C. molle* corresponds to their known geographical ranges, the potentially available habitats of *C. dickinsonianum* are distant from its known populations. As postulated in previous studies (Hernandez et al., 2006; Wisz et al., 2008; Merow, Smith & Silander Jr, 2013; Boria et al., 2014; Fourcade et al., 2014), we applied numerous methods to obtain the most reliable models, including reducing sampling bias, excluding correlated climatic variables and performing experiments with regularization multiplier values. Unfortunately, this approach was not effective in the case of endemic *C. dickinsonianum*, known so far from only three localities. Apparently, the lowest number of localities required to produce reliable models using the Maxent application is four—this amount of occurrence data was sufficient to obtain satisfactory maps of the suitable habitat distribution of *C. molle.* Another, less plausible explanation for the *C. dickinsonianum* model overfitting, is the existence of some climatic factor not included in the analysis or ecological relationships that prevented the migration of *C. dickinsonianum* from southern Chiapas to other areas.

It appears that the evolutionary success of the family Orchidaceae (ca 30,000 species) can be connected with the possibility to create hordes of hybrids, which can colonize new habitats. It may lead to origination of new species. The new hybrid lines that are not subject to introgression, have two genomes that have different evolutionary histories. These populations, as a result of random events and selection may lose some alleles, thus leading to a genetic patchwork but with a predominance of the genome of one of the ancestors. As a result, works on orchid phylogeny carry a very high risk of error. In particular, this concerns works based solely on plastid markers (plants barcoding DNA), which are inherited in the maternal line, and the ITS, which is a multi-copy marker being a subject to concerted evolution easily leading to the elimination of one of the parental copies. Consequently, we can observe a species whose morphological traits (resulting from nuclear genes) are in conflict with the above markers.

##  Supplemental Information

10.7717/peerj.4162/supp-1 Data S1 Alignment of ITS of analysed taxa

10.7717/peerj.4162/supp-2 Data S2 Alignment of XDH gene of analysed taxa

10.7717/peerj.4162/supp-3 Data S3 Alignment of matK gene of analysed taxa

10.7717/peerj.4162/supp-4 File S1 Chromatogram files (.abi) of hybrid

10.7717/peerj.4162/supp-5 Data S4 Complete database of localities of *C*. *irapeanum*, *C*. *molle* and *C. dickinsonianum* gathered during the studies

10.7717/peerj.4162/supp-6 Data S5 Spatial-filtered dataset of localities of *C. irapeanum*, *C. molle* and *C. dickinsonianum* used in the ENM analysis

10.7717/peerj.4162/supp-7 Figure S1 Frequency of the ovules contained embryos or ovules that were unfertilized within four ovaries of *C. × fred-mulleri* No. –number of the ovary; n, number of ovules analyzed in each ovary; standard error, bars.

## Additional Information and Declarations

### Data Availability

The following information was supplied regarding data availability: The raw data has been provided as a Supplemental File.

### New Species Registration

The following information was supplied regarding the registration of a newly described species: *Cypripedium* × *fred-mulleri* Szlach., Kolan. & Górniak, hybr. nat. nov. LSID 77173689-1.
