# Supplementary figures and images for: First Guatemalan record of natural hybridisation between Neotropical species of the Lady’s Slipper orchid (Orchidaceae, Cypripedioideae)
Source: PeerJ. 2017 Dec 22;5:e4162. doi: 10.7717/peerj.4162 (PMC5742529; doi:10.7717/peerj.4162)

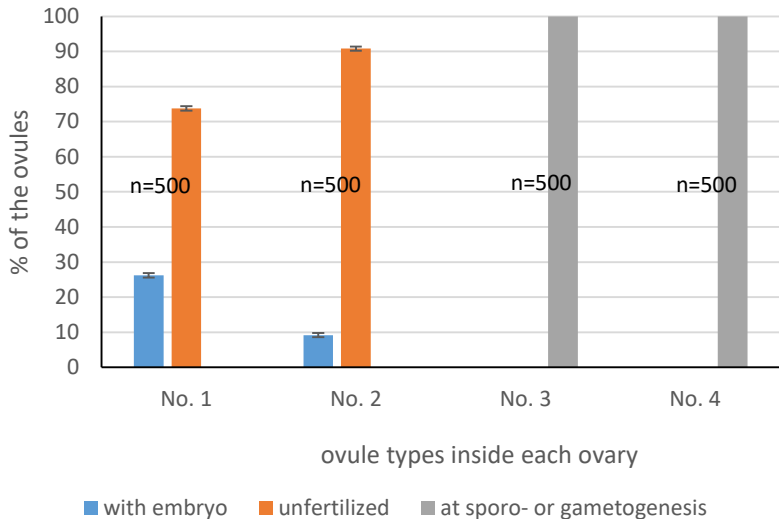

Supplement: Figure S1 — No. –number of the ovary; n, number of ovules analyzed in each ovary; standard error, bars. [file peerj-05-4162-s007.pdf]
